# Supplementary material for: Enhancing human aspects of care with young people with muscular dystrophy: An evaluation of a participatory qualitative study with clinicians
Source: PLoS One. 2022 Feb 25;17(2):e0263956. doi: 10.1371/journal.pone.0263956 (PMC8880938; doi:10.1371/journal.pone.0263956)
Supplement: S1 Appendix — (DOCX) [file pone.0263956.s001.docx]

**Appendix A: Abbreviated list of recommendations (site 1)**

**Routine clinical processes**

- Consider flexibility in frequency, length, and focus of clinic visits
  - Flexibly prioritise which clinician sees families each visit (family input?)
  - Some shorter focused visits with 1 to 3 key clinicians
  - Some visits focused primarily on psychosocial care
- Ensure adequate time for pre-clinic rounds to prioritise integrated, streamlined care
- Develop ‘safe spaces’ for sociocultural aspects of care
  - Create physical therapeutic space (separate from other clinic spaces)
  - Options for separate appointments (outside of clinic)
- Create mechanisms to convey psychosocial priority to families
  - Modify standard assessment forms and procedures to address human needs
- Develop processes for determining if/when child or caregiver(s) is or is not present
  - Consider processes for when child is taken out of room (e.g. pre-determine time)
  - Child/parent time out of clinic as standard part of visit (e.g. with social worker).
  - Identify when caregiver/child needs a break during clinic
- Create mechanisms for peer to peer support (create community)
  - Promote advocacy/support groups
  - Create opportunities for families to meet
    - MD only clinic day
    - Central space in waiting area for families to connect
    - Speakers on MD including socio-political aspect of living with MD
    - Team camp
    - ‘Meet ups’ based on young people’s interests – not just “therapy” (anime group, remote videogame tournaments, etc)

**Interpersonal interactions**

- Create culture for expressing and supporting negative emotions (eg grief, anger)
  - Skill building and reflective practice to enhance clinician ability to recognize and respond to parent/child’s needs to express/discuss a variety of emotions/issues.
- Create mechanisms to convey psychosocial priority to families
  - Develop related ‘mission statement’ for clinic brochure & online
- Strategies for addressing psychosocial needs of staff
- Spiritual aspects of MD journeys
  - Incorporate queries re spirituality into supportive conversations
- Upskill clinicians on socio-political aspects of living with MD:
  - upskilling clinicians on disability stigma, marginalisation
  - referral to resources (eg blogs, films, TV)
  - cultural appropriateness

**Staffing allocations**

- Shift staffing focus to ‘human’ aspects of care:
  - Re-frame Social Worker role
    - Reallocate funding discussions to other personnel
    - Present role to families as focused on psychosocial care
  - Increase FTE of social worker, rec therapist and psychologist
  - Incorporate psychosocial strategies into all clinician practices
  - Upskill clinicians on psychosocial, existential and spiritual aspects of MD
  - Hospital multi-faith chaplain or pastoral care

**Ongoing dialogues/critical reflexivity**

- Regular critical reflexivity in team meetings
- Check in with families outside of clinic regarding recent clinic visit experiences
- Develop knowledge of language effects and alternate approaches to conveying partnership
- Identify external facilitation opportunities
- Identify uses for theory/frameworks
