# Supplementary material for: Enhancing human aspects of care with young people with muscular dystrophy: An evaluation of a participatory qualitative study with clinicians
Source: PLoS One. 2022 Feb 25;17(2):e0263956. doi: 10.1371/journal.pone.0263956 (PMC8880938; doi:10.1371/journal.pone.0263956)
Supplement: S2 Appendix — (DOCX) [file pone.0263956.s002.docx]

**Appendix B Abbreviated list of recommendations (site 2)**

**Clinic Organisation**

- Maintain continuity of clinicians where possible
- Work to reduce interruptions
  - Don’t enter the room uninvited
  - Maintain current process of clinicians visiting patients and families (who stay in a single appointment room for much of the appointment)
  - Sign on the door with who is in the room (don’t let anyone close the door)
- Extend allocation of appointment times beyond medical team to other health professionals so they will have sufficient time with patients and families
- Create communication methods to reduce repetition of assessment and advice, to improve quality of patient experience and reduce mixed messages
  - For learners – better direction from staff physicians; get the learners to read history before
  - Have a one page primer about the condition and the child – expectation should be there that they know about that condition before they come to clinic
  - Sometimes trainees have to learn by observation for consideration for the family
- Develop a strategy around when children should be in the room
- Consider asking families in advance of clinic about topics important to them to cover
- Consider a written summary of advice for post-visit for the family (which the team can also review)

**Team communication**

- Create team communication mechanisms for in-the-clinic, and post-appointments
- Ensure pre-clinic and in-the-clinic communication methods mention ‘sensitive’ topics (and who will discuss them)
- Seek efficient ways to provide team information on the family

**Enhance prioritisation of ‘human’ aspects**

- Develop methods to better understand family’s values and visions as they evolve over the child’s life with exposure to different ways of understanding disability and emerging emotional, social, and moral challenges.
- Keep working to bring the child and family’s life into view, even when talking about the body.
- Develop methods by which team members are (more) aware of home treatment routines, and the impact they have on the child and family’s quality of life.
- Help parents and young people develop positive ways of understanding and ‘accepting’ disability (ensure a clinical routine to do so)
- Develop processes for clinicians to continue to deepen reflection on clinic processes and impacts on patients
- Team development time opportunities (e.g. retreats, regular team check-in/reflection times)

**Interpersonal interactions**

- Skill development in counselling/support strategies, to support self-management effectively and non-judgmentally.
  - Professional development opportunities (e.g. posing open-ended questions; mentoring and feedback from skilled colleagues)
- Related to this, working to reframe what are often perceived of in health care as ‘bad choices’; approaching with curiosity

**Institutional constraints**

- Creating a dedicated physical space for team to communicate while clinic is in session (advocating for space for whole team)
