# Supplementary material for: Enhancing human aspects of care with young people with muscular dystrophy: An evaluation of a participatory qualitative study with clinicians
Source: PLoS One. 2022 Feb 25;17(2):e0263956. doi: 10.1371/journal.pone.0263956 (PMC8880938; doi:10.1371/journal.pone.0263956)
Supplement: Attachment — Submitted filename: Reviewers comments.doc [file pone.0263956.s003.doc]

The study evaluated a participatory interventional procedure geared towards enhancing ‘human’ aspects of MD management among clinicians. The study is relevant to literature and seems to have sound methodologies. However, the blinding of the previous studies with the full reports on the methodologies limits a thorough appraisal of the study’s materials and methods. Shallow explanation of these ‘human’ aspects of care under the ‘introduction’ section of the study failed to highlight the relevance of these aspects in the management of MDs. Below are specific comments.

**Title**: This is apt for the study.

**Abstract**

1. Purpose: Is it entirely correct to call this study a ‘3-year’ study considering the fact that the study lasted 1.5 years in one of the two centres?
2. Purpose: The last sentence under this section looks more like a ‘methods’ statement rather than a ‘purpose’ statement.
3. Results: As a follow-up, can the authors list ‘why’ there were variations in the ways clinicians changed their thinking and practices?

**Introduction**:

1. The first statement under the introduction was the aim of the study. This is uncommon and unfamiliar in the scientific world. This may be more apt as the concluding statement of the last paragraph under introduction, which the authors had already done.
2. Assessing and improving the ‘human’ aspects of care is the focus of this study. Despite this, authors failed to elaborate on these aspects of care under the introduction. Authors may also need to highlight that these parameters are common and are usually neglected in MD management. This will help to better justify the study.

**Materials and Methods**

1. Authors reported that the methodologies of the present study had been reported elsewhere (references number 5 and 14). However, these references were blinded thereby making it difficult for the reviewer to consult these articles. Can the authors please unblind the blinded references in order to facilitate better review process?
2. ***The Intervention:*** How many series of the 2-hour meetings were conducted in each case? What are the spacings (time lag) between two consecutive interventions? These questions were probably answered in the previous publications but there was no way the reviewer could ascertain that since the publications were blinded. Though these were mentioned under the results, they are supposed to be talked about under methodology.
3. ***Study sites and participants:*** The sentence ‘Most significantly, the study duration was considerably longer at site 1 versus site 2 (3 and 1.5 years respectively)’ seems confusing. Authors should please consider rephrasing the sentence.
4. With the reported difference in the duration of the study at the two sites, it is logical to think that some procedures were shelved for site 2. How did the procedures (especially the interventions) in the two sites differ? What precautions were taken to make sure that the differences in procedures did not affect the validity of the study?

**Results**

1. Detailed and reported in a coherent manner.
2. Authors cited table 3 before table 2 intext. Reverse should be the case. Alternatively, authors can redesignate the tables accordingly.
3. Table 2: There is a repetition in the cell where the second column intersects with the second row. Please correct: “……clinicians attended each dialogue at site 1, and a mean of and a mean of 9 clinicians…..”
4. I am just thinking if this quote on page 11 not better suited for the third sub-question (item 1.3) instead of the first:

It doesn't give people the sense of working collaboratively. … People are called to the table when there's an actual issue, and do come. But on a regular ongoing basis, I think it's hit and miss.

5. Page 24: The word ‘to’ before the phrase “do what is best” needs to be expunged from the quote. The affected aspect of the quote is shown below:

‘…..One clinician then added that they should to “do what is best” for the family and….’

**Discussion**: Well-written
