# Supplementary material for: Enhancing human aspects of care with young people with muscular dystrophy: An evaluation of a participatory qualitative study with clinicians
Source: PLoS One. 2022 Feb 25;17(2):e0263956. doi: 10.1371/journal.pone.0263956 (PMC8880938; doi:10.1371/journal.pone.0263956)
Supplement: Attachment — Submitted filename: Response to Reviewers.docx [file pone.0263956.s004.docx]

**Response to Editor and reviewer comments**

Thank you to the Editor and reviewers for their input into our manuscript. We have made significant changes to the paper as a result, particularly the Introduction and Materials and Methods sections. We believe the manuscript is considerably stronger as a result.

**Responses to Editor comments**

1. As an Editor I feel it will be useful for both reviewers if you can unbind the previous studies with the full reports on the methodologies so that reviewers will have a thorough appraisal of the study’s methods.

**Author response:** The study has been unblinded so that the reviewers have access to previous studies.

2. Also provide more detail explanation of the ‘human’ aspects of care under the ‘introduction’ section of the study to further highlight the relevance of these aspects in the management of MDs.

**Author response:** Thank you. We have added considerably more detail about this concept. Please see our response to reviewer 1’s second comment on our introduction section.

3. In as much I do not want to the authors to write a lay paper I will agree with reviewer 2 to edit/simplify the paper in places so that complex information and messages comes across to readers in a clear and understandable way.

**Author response:** We have completed a thorough re-read and simplified the paper where possible/needed. This has principally been in the Introduction, Method and Discussion sections. The reviewers had said that the Results reporting was clear so we did little editing there.

4. I will also want to see clarifications about the methodology from an RE perspective and explicit use of the terminologies and language of RE or a clearification that the study was not a true RE. Can the authirs address while initial theory was not laid out. Perhaps some of the questions can be answered if reviewers have access to the method proper.

**Author response:** We have addressed this question in detail below to clarify that we only *drew from* elements of RE. The reviewers now have access to our two related papers which outline the methodology and theoretical underpinnings of the original research in more detail.

*5. Journal Requirements:* Please review your reference list to ensure that it is complete and correct. If you have cited papers that have been retracted, please include the rationale for doing so in the manuscript text, or remove these references and replace them with relevant current references. Any changes to the reference list should be mentioned in the rebuttal letter that accompanies your revised manuscript. If you need to cite a retracted article, indicate the article’s retracted status in the References list and also include a citation and full reference for the retraction notice.

**Author response:** There are no retracted references.

6. Please ensure that your manuscript meets PLOS ONE's style requirements, including those for file naming.

**Author response:** We have updated the style to match Plos One’s requirements as requested, including placing the tables within the text.

7.  Please ensure that you include a title page within your main document. We do appreciate that you have a title page document uploaded as a separate file, however, as per our author guidelines (<http://journals.plos.org/plosone/s/submission-guidelines#loc-title-page>) we do require this to be part of the manuscript file itself and not uploaded separately. Could you therefore please include the title page into the beginning of your manuscript file itself, listing all authors and affiliations.

**Author response:** This change has been made.

8. Thank you for including your ethics statement: "The human research ethic boards from both study sites approved the study. Holland Bloorview: 17-729 CHEO: 17-117X Written consent was obtained from all participants." Please amend your current ethics statement to include the full name of the ethics committee/institutional review board(s) that approved your specific study. Once you have amended this/these statement(s) in the Methods section of the manuscript, please add the same text to the “Ethics Statement” field of the submission form (via “Edit Submission”).

**Author response:** We are able to provide full ethics committee names to the journal. We have added the text to the Ethics Statement field of the journal as requested.

9. Please ensure you include additional information regarding the survey or questionnaire used in the study and ensure that you have provided sufficient details that others could replicate the analyses. For instance, if you developed a questionnaire as part of this study and it is not under a copyright more restrictive than CC-BY, please include a copy, in both the original language and English, as Supporting Information, or include a citation if it has been published previously.

**Author response:** No survey or questionnaire was used in this study.

10. We note that you have indicated that data from this study are available upon request. PLOS only allows data to be available upon request if there are legal or ethical restrictions on sharing data publicly. For information on unacceptable data access restrictions, please see <http://journals.plos.org/plosone/s/data-availability#loc-unacceptable-data-access-restrictions>. In your revised cover letter, please address the following prompts:

a) If there are ethical or legal restrictions on sharing a de-identified data set, please explain them in detail (e.g., data contain potentially identifying or sensitive patient information) and who has imposed them (e.g., an ethics committee). Please also provide contact information for a data access committee, ethics committee, or other institutional body to which data requests may be sent.

b) If there are no restrictions, please upload the minimal anonymized data set necessary to replicate your study findings as either Supporting Information files or to a stable, public repository and provide us with the relevant URLs, DOIs, or accession numbers. Please see <http://www.bmj.com/content/340/bmj.c181.long> for guidelines on how to de-identify and prepare clinical data for publication. For a list of acceptable repositories, please see <http://journals.plos.org/plosone/s/data-availability#loc-recommended-repositories>.

We will update your Data Availability statement on your behalf to reflect the information you provide.

**Author response:** There are ethical restrictions on sharing the de-identified data set because it contains potentially identifying or sensitive patient information. Participant confidentiality is a requirement of the ethic committee. We have included more detailed information in our cover letter and during email correspondence with the journal.

**Responses to reviewers' comments**

Reviewer #1:

**Reviewer 1 Comment 1:** The study evaluated a participatory interventional procedure geared towards enhancing ‘human’ aspects of MD management among clinicians. The study is relevant to literature and seems to have sound methodologies. However, the blinding of the previous studies with the full reports on the methodologies limits a thorough appraisal of the study’s materials and methods. Shallow explanation of these ‘human’ aspects of care under the ‘introduction’ section of the study failed to highlight the relevance of these aspects in the management of MDs. Below are specific comments.

**Author response:** Thank you for your positive comments. These references have now been unblinded. We have added considerably more information about the human aspects of care and their relevance to MD management. We explain those changes in detail below in response to this reviewer’s comment 7).

**Reviewer 1 Comment 2:** Title: This is apt for the study.

**Author response:** Thank you.

Abstract
**Reviewer 1 Comment 3:** Purpose: Is it entirely correct to call this study a ‘3-year’ study considering the fact that the study lasted 1.5 years in one of the two centres?

**Author response:** We believe it is appropriate to describe the project as a 3-year study across two sites as the empirical work at each site fed into that at the other and the project overall. However, to avoid any possible confusion, we have removed the time descriptor from the abstract so that the study length at each site is introduced in the methods section where we can clarify how long was spent at each site.

To provide an overarching understanding of the length of the project we now introduce the timeframe at the beginning of the methods as a “**3-year** collaboration between researchers, clinicians and young people…” (page 4, line 24) and then clarify the length of time at each site on page 7 (lines 20-22) as follows: “**T**he study duration was considerably longer at site **1 (3 years) than** at site 2 (1.5 years) **and there were half** the number of dialogues and approximately one quarter the number of observations at site 2.” [new text in bold]

**Reviewer 1 Comment 4:** Purpose: The last sentence under this section looks more like a ‘methods’ statement rather than a ‘purpose’ statement.

**Author response:** This sentence has been moved to the methods.

**Reviewer 1 Comment 5:** Results: As a follow-up, can the authors list ‘why’ there were variations in the ways clinicians changed their thinking and practices?

**Author response:** Yes, great idea. We have added a sentence to allude to some of the reasons for these variations. The new sentence is in bold below.

Page 2, lines 15-18: “Not all clinicians changed their thinking and practices in the same ways, or to the same extent, and there were differences between the sites. **These differences were likely due to numerous factors including varying levels of clinician comfort with examining and shifting their own practices, and differing formal and informal clinic routines at each site**.”

Introduction:
**Reviewer 1 Comment 6:** The first statement under the introduction was the aim of the study. This is uncommon and unfamiliar in the scientific world. This may be more apt as the concluding statement of the last paragraph under introduction, which the authors had already done.

**Author response:** We agree that this type of introduction is less common in the biomedical scientific world, but not so in social *science*. Our research team spans both. Although this journal is likely to have readers from both disciplines, we have rephrased the introduction as the reviewer has suggested in the process of simplifying our argument in response to other reviewer comments. This has included removing the first sentence (page 3, line 2), and reworking the final paragraph of the introduction as below [new text in bold]:

Page 4, lines 13- 19: In response to these challenges, **we developed a project that aimed to**: 1) examine the assumptions unpinning clinical practice; 2) **create adaptable recommendations for change** to ensure context-specific relevance; and 3) change multiple aspects of clinical practice towards enhancing the human aspects of living with MD (5,20). This paper reports on the impact of the **interventional aspects of that project** by responding to the following research question: How and why did the **project** change clinician **thinking and** practices in relation to the human aspects of care in outpatient neuromuscular clinics?

**Reviewer 1 Comment 7:** Assessing and improving the ‘human’ aspects of care is the focus of this study. Despite this, authors failed to elaborate on these aspects of care under the introduction. Authors may also need to highlight that these parameters are common and are usually neglected in MD management. This will help to better justify the study.

**Author response:** We have elaborated on the concept of human aspects of care in the first paragraph of the introduction as below.

Page 3, lines 12-23: “At times, this has come at the cost of the ‘human’ aspects **of care. We use the term “human” as a shorthand that extends beyond “psychosocial” concerns to capture the emotional, existential, social, and moral dimensions of illness experiences and care that co-exist with the physical/biological and technical dimensions (4, 5). For example, human aspects include** dealing with disability stigma, creating meaningful shortened lives, and managing family interactions (4,5). **The human dimensions of living with MD overlap with and extend the concept of person-centred care, which, at least in practice, tends to focus on autonomy and decision making (6,7). Our conceptualisation of the human aspects co-exists with biomedical dimensions and is interrelated with them. Attending to these human dimensions aligns with arguments that extending life, or enhancing participation of people with MD in society, should not be the only foci: an enriching and enjoyable life (regardless of its length) is also highly salient.**”

Materials and Methods
**Reviewer 1 Comment 8:** Authors reported that the methodologies of the present study had been reported elsewhere (references number 5 and 14). However, these references were blinded thereby making it difficult for the reviewer to consult these articles. Can the authors please unblind the blinded references in order to facilitate better review process?

**Author response:** The study has now been unblinded including the two key references which provide more detail on the methodology underpinning the study.

**Reviewer 1 Comment 9:** The Intervention: How many series of the 2-hour meetings were conducted in each case? What are the spacings (time lag) between two consecutive interventions? These questions were probably answered in the previous publications but there was no way the reviewer could ascertain that since the publications were blinded. Though these were mentioned under the results, they are supposed to be talked about under methodology.

**Author response:** Thank you to the reviewer for raising this point. We are aware that there is debate about the correct place to put participant numbers with some platforms now insisting that they are only reported in the results section. We are fine either way, but have decided leave these numbers in the results because we believe they are relevant to the evaluation of the ‘success’ or otherwise of our intervention (i.e., more related to the results than the methods). The numbers of dialogues were not predetermined a-priori and whether we were able to organise a sufficient number of dialogues (and how well they were attended) was also relevant to the success of the project. However, in response to this comment, and to give readers an early sense of the frequency of the dialogues, we have added an indication of the frequency of the dialogues to the methods (new text in bold):

Page 7, lines 1-2: “The key intervention strategies were a series of **approximately quarterly**, 2-hour meetings (“dialogues”) between the researchers and participating clinicians at each site.”

**Reviewer 1 Comment 10:** Study sites and participants: The sentence ‘Most significantly, the study duration was considerably longer at site 1 versus site 2 (3 and 1.5 years respectively)’ seems confusing. Authors should please consider rephrasing the sentence.

**Author response:** The sentence has been rephrased. It now reads: “**T**he study duration was considerably longer at site 1 **(3 years) than at** site 2 (1.5 years). **There were** half the number of dialogues and approximately one quarter the number of observations at site 2.” (page 7, lines 20-22)

**Reviewer 1 Comment 11:** With the reported difference in the duration of the study at the two sites, it is logical to think that some procedures were shelved for site 2. How did the procedures (especially the interventions) in the two sites differ? What precautions were taken to make sure that the differences in procedures did not affect the validity of the study?

**Author response:**  Thank you to the reviewer for requesting further detail here. To be clear, we are not attempting to say that the study was the same at both sites. In contrast to more conventional study designs, the explicit intention of this research was to create a study design that was adapted to each site. We discuss the variations in procedures in each site in detail in the results section. “Validity” refers to the degree to which research findings accurately reflects or assesses the specific concept that the researcher is attempting to measure. Validity in *quantitative* research is usually approached by controlling more variables or reducing possibilities of “bias”. Most qualitative research does not try to control for such factors. We have been explicit about this and have been careful to avoid any claims that the results at each site, and any comparisons we might make between them, are definitive ‘truths’. For example, we state in the Method and Materials:

Page 6, lines 6-8: “**Aligned with our critical social science approach to the project,** the underlying assumption of **RE** is that there is no final truth/knowledge but that it is possible to work towards a closer understanding of what is happening”

And in the Discussion (page 35, lines 13-16): “When considering the implications of this study, it is important to be aware of the conceptual basis of a realist evaluation, **our theoretical underpinnings** and the claims they support. This type of **project and** evaluation does not attempt to find a stable or singular truth, but to enhance understandings of how and why an intervention might work (or not work).”

NB: both statements have been modified slightly in response to an unrelated question about our use of Realist Evaluation by Reviewer 2 (Comment 3).

Results
**Reviewer 1 Comment 12:** Detailed and reported in a coherent manner.

**Author response:** Thank you.

**Reviewer 1 Comment 13:** Authors cited table 3 before table 2 intext. Reverse should be the case. Alternatively, authors can redesignate the tables accordingly.

**Author response:** Our apologies for this oversight. The tables are now cited and displayed in the correct order. They are also now placed within the body of the manuscript, as per the journal’s requested layout.

**Reviewer 1 Comment 14:** Table 2: There is a repetition in the cell where the second column intersects with the second row. Please correct: “……clinicians attended each dialogue at site 1, and a mean of and a mean of 9 clinicians…..”

**Author response:** Thank you for noting this typographical error. This has now been corrected - the second point now reads: “All clinicians attended at least one dialogue and a mean of 9 (62%: range 43-77%) attended each dialogue at site 1, and a mean of 9 (43%: range 35-50%) at site 2” (Table 3, page 13).

**Reviewer 1 Comment 15:** I am just thinking if this quote on page 11 not better suited for the third sub-question (item 1.3) instead of the first:
It doesn't give people the sense of working collaboratively. … People are called to the table when there's an actual issue, and do come. But on a regular ongoing basis, I think it's hit and miss.

**Author response:** We agree, and have now moved this quote, and the sentences introducing it, to page 21, lines 1-7.

**Reviewer 1 Comment 16:** Page 24: The word ‘to’ before the phrase “do what is best” needs to be expunged from the quote. The affected aspect of the quote is shown below:
‘…..One clinician then added that they should to “do what is best” for the family and….’

**Author response:** Thank you - ‘to’ has been expunged as suggested.

**Reviewer 1 Comment 17:** Discussion: Well-written

**Author response:** Thank you.

Reviewer #2
OVERALL FEEDBACK:
**Reviewer 2 Comment 1:** Thank you very much for the opportunity to review this manuscript. I would like to congratulate the authors on an extremely insightful study that I think has wide applicability, well beyond children and youth with MD. I have tried to provide thoughtful and constructive feedback that I hope will improve the impact of the manuscript. I have two main points of general feedback, followed by specific feedback broken down by section.

**Author response:** Many thanks to the reviewer for their positive comments on our research, including its possibilities for wide applicability. We have responded to the reviewer’s comments below.

GENERAL FEEDBACK:
**Reviewer 2 Comment 2:** The manuscript is very well written overall. However, at times it is a lot. There is NO DOUBT that this group of authors are extremely deep and complex thinkers. But an important skill is being able to take a lot of complex information and distill it in a clear and understandable way. I wonder if the authors could re-read and edit the paper with this in mind. When I was doing my PhD, my supervisor used to say, “be kind to the reader”. For example, I personally love Bourdieu and agree that his thinking and philosophical concepts can provide a relevant lens through which to view theory and practice in rehabilitation. However, given the density of the manuscript I think the authors can make the point about practice change in a way that is much more succinct and accessible, thereby paring down the intensity and length of the manuscript.

**Author response:** Thank you to the reviewer for this comment. We have made a thorough edit of the manuscript to simplify concepts where possible. We agree that there is a lot in this paper. We have revised it with this in mind. We have simplified discussion of practice change, now only implicitly referring to Bourdieu’s concepts, while retaining the citations so that readers can follow where we have derived the ideas if desired. We hope we have been kind, to both novice and experienced readers.

The following is an example of the changes we have made throughout as well as a specific response to the comment about Bourdieu:

Page 3, line 24, to page 4, line 7 [new text in bold]: “Even **with** clear parameters to change, such as applying clinical guidelines, **multidisciplinary clinicians’** attempts at change are often **limited or** ineffective [8]. **Many factors influence the ability to change including systemic constraints, cultural norms and the variability of what is required for change across contexts [8]. In their review of primary care, Lau, Stevenson [7] stated: “Implementation of any type of intervention is complex, dynamic and influenced by a variety of factors at the level of external context, organisation, professional and intervention” (p. 37).** There are deeply ingrained ways of acting, thinking and doing that are formed through repetition and internalisation of social norms [9z.

**Reviewer 2 Comment 3:** The authors report using a realist evaluation (RE). I do see RE imbued in the methodology, including the way that the research question is written. However, there are almost NO explicit talk about RE, no talk about context, mechanism, and outcomes, and context-mechanism-outcome configurations, nothing about initial program theories etc. etc. I see it more clearly in some respects but not in others. For example, typically RE begins by creating an initial program theory (drawing on program architects and/or realist syntheses) which consists of several C-M-O configurations. Then, the initial program theory is tested via the research, then refined in response to the research. The authors describe the four steps in their data analysis (i.e., Step #1-provisional analysis of all data, Step #2, discussion and advance of the preliminary analysis), but as the reader I am wondering “Does this mean they created an IPT?” If so, how, specifically? Despite the authors referring to RE and some of the characteristics of RE being evident to a trained eye, the methodology from an RE perspective is opaque. I wonder why the authors chose not to be explicit and use the language of RE. This approach could undermine the veracity of the claim that this was an RE study and therefore ultimately undermine the quality of the work. Although I do not doubt the quality, I suggest that if it is an RE, be explicit. If it was not a true RE then be explicit about that as well, refrain from calling it an RE, and decide on a more accurate term. The other associated issue is that I feel that RE has a huge potential to contribute to the development of knowledge in rehabilitation. But, if as authors and researchers we are opaque, how can we advance the use of this methodology? Part of the contribution of this paper are the findings related to MD, to be sure, but a big contribution could also be in demonstrating and providing an exemplar of the use of RE in rehabilitation.

**Author response:** Thank you to the reviewer for raising this question. This study was not intended to be a classical RE study but rather, in the spirit of methodological ‘bricolage’, we drew from RE methods to address the study’s aims and epistemological commitments. In doing so, we were keen to keep theoretical congruency between our research, which was derived from post-structuralist and critical approaches, and this evaluation. As our underpinning theories were critical to our understanding of the issues in the clinics, and to our evaluation of the changes the clinicians/clinics made, we looked for a non-positivist and flexible evaluative approach that allowed us to undertake rich, context specific analysis of structures, processes and effects. In this vein, we were keen to avoid epistemological and methodological rigidity which might constrain our analyses and reporting. Thus, we drew on RE, admittedly in a much-adapted (less constrained) form. Further, as we only applied the evaluation post-hoc we did not use the iterative elements of RE (such as initial program theory with C-M-O configurations). Importantly, we believe this approach has been able to produce valuable, insightful and thorough insights (as this reviewer has mentioned).

We have made a number of changes in the manuscript to clarify that we did not directly use a realist evaluation, but drew from some of its key tenets and epistemological/methodological elements. These changes have included removing the term ‘realist’ from the paper’s title, and reducing the strength of our claims to our use of RE throughout the manuscript. For example (new text in bold):

Abstract: Page 2, lines 8-9: “We **drew from** realist evaluation approach**es** to assess changes in clinical care associated with the intervention.”

Method: Page 5, line 21 to page 6, line 21: “**To be consistent** with the critical social science underpinnings of our study, **we sought evaluation approaches that conceptually aligned with the study. After considering a number of evaluative approaches,** we drew from elements of a realist approach. Realist evaluation approaches (RE) considers the complexities of an intervention through a focus on improving understandings of how and why interventions work or do not work in particular contexts (21). RE is underpinned by the premise that causation is not easily accessed within a complex system and cannot usually be directly observed, but can be investigated. RE considers both material and social worlds as ‘real’ in that they can have ‘real effects’ (21). Material environments, clinical tools, measuring devices and clinical practices, as well as programs, policies, social institutions are considered to have an impact on how and whether an intervention works**. Like our critical social science approach to the project**, the underlying assumption of a realist evaluation is that there is no final truth/knowledge but that it is possible to work towards a closer understanding of what is happening.

We draw in particular from the Realist Evaluation Framework which has been increasingly and diversely applied in healthcare environments (e.g., 22, 23). Following realist principles, the **framework** supports an analysis of the ways in which context affects how, and for whom, an intervention works – thus acknowledging that observed outcomes are not simply the result of the intervention but of how it interacts with elements of the context (24). Our approach drew on **some of** its principles **(particularly from its ‘outcome elements’ which focus on who the intervention works for, and how the intervention application and context affect its impact)** but used a flexible design in keeping with the project goals and theoretical groundings. **Our analysis was only applied post-hoc so did not include iterative elements of a RE (no Initial Program Theory).** Our evaluation **approach** was discussed and refined by members of the research team (initials blinded for review) to develop evaluative questions from the ‘overarching and subsidiary’ questions delineated by Westhorp (21).

INTRODUCTION:
**Reviewer 2 Comment 4:** The introduction would be strengthened if the authors made clear, from the opening sentence, what kind of intervention they are referring to. The first line states, “This paper evaluates an intervention” then throughout the paragraph the researchers talk about “health care” practice “clinical practice”, an “intervention in clinical sites” and after reading the whole introduction I, as the reader, still had no idea about what the intervention is and what type of health care professionals are involved. In addition, several of the references cited in the introduction relating to “clinical practice” broadly (i.e., #7, 13) actually relate to primary care and mostly physicians in primary care (as per references #7 and #13). It is recommended that the authors clarify what type of intervention they are referring to from the very beginning AND include references, where possible, from the body of literature of all the professionals involved that support to the content of their introduction, not just primary care (physicians).

**Author response:** In response to this comment about the lack of clarity regarding the intervention in the introduction, and that from the first reviewer (see: reviewer 1, comment 6) about the formatting of the introduction, we revised the introduction into a more traditional biomedical journal format where the intervention is introduced in the Method and Materials section. This means that we are able to discuss the complexities and novelty of the intervention in more detail with is less room for confusion. In response to this reviewer’s comments about the breadth of literature, we have broadened our citations to include those from other professions, so that when the multidisciplinary teams are discussed in the methods section, this makes more sense.

**Reviewer 2 Comment 5:** As stated in #1 above, the paragraph from “Clinical practices” ending in “contextually relevant” could be very easily simplified and shortened by tightening the writing.

**Author response:** This paragraph has been changed in response to this reviewer’s comments. The paragraph now starts on page 3, line 24 and its new wording is reproduced below in response to this reviewer’s next comment.

**Reviewer 2 Comment 6:** For the sentence in the introduction that states “Even when there are clear parameters…” and ends in “…in part because what is required to implement changes in one context varies from the next [8]. I understand why, given that the authors are building towards rationalizing the methodology (RE), the focus on context. However, the sentence feels unbalanced given that there have been many documented reasons (across health care professions) why practice change is difficult. So, to be fair-minded and balanced, I suggest that the authors indicate, even in parentheses, that there are many documented reasons why practice change is difficult (e.g., ) and after the change is made in #1 above, use citations that are relevant to the fields of practice under study. THEN, comment on differing contexts being one such important factor. Also, given that the study is a RE, consider including a reference from the RE literature to support this point. As stated above this whole paragraph can be simplified while still making the points that (a) practice change is hard to shift for many reasons, (b) one of the main reasons relates to the varying contexts within which programs are designed then subsequently delivered.

**Author response:** We take your point, it wasn’t our intention to point only to context as the cause of difficulties, but rather simply that context is always a factor. We have broadened our discussion to speak to more of the challenges as suggested. The paragraph has been changed considerably in response to this comment and this reviewer’s other point about simplification.

Page 3, line 24 to page 4, line 12: Clinical practices are difficult to shift (8). Even **with** clear parameters to change, such as applying clinical guidelines, **multidisciplinary teams’** attempts at change are often **limited or** **ineffective (9). Many factors influence the ability to change, including systemic constraints (10), cultural norms (11, 12) and the variability of what is required for change across contexts (9, 13). In their review of primary care, Lau, Stevenson (14) stated: “Implementation of any type of intervention is complex, dynamic and influenced by a variety of factors at the level of external context, organisation, professional and intervention” (p. 37). There** are deeply ingrained ways of acting, thinking and doing that are formed through repetition and internalisation of social norms (15). These learned habits are often mistaken as inherent or natural (16), including in the context of disability healthcare and MD (17)**, and are thus** very hard to change or even notice. As a result, it is not surprising that there is a general agreement in the literature that to successfully change clinical practice (which has multiple habitual practices, values and assumptions), interventions need to be sustained and repeated, and be contextually relevant (14, 18, 19).

MATERIALS and METHODS:
**Reviewer 2 Comment 7:** The authors refer to “dialogical exchange”. When I read “dialogical” I immediately think “Bhaktin”. Then, there is no description about what is meant by a “dialogical exchange” or reference provided. What are the specific criteria or parameters that made it a “dialogical exchange” versus a discussion or interview? Please be clear, perhaps adding a reference, b/c I am left wondering what the guiding lens was for the interaction between the researchers and the clinicians at each site. I apologize if this information is reported elsewhere in other publications detailing the project methodology. The references have been blinded so I cannot cross-reference. In any case, I think it is worth clarifying here, at least with a reference, to make it more understandable to the reader, particularly the reader from a non-academic audience.

This concern came up again under the section titled “The Intervention”. It seems the dialogues are part interview, part mentorship and coaching. Please add a reference for what was done or an explanation of how the “dialogical protocol” was determined and how it was anticipated that this particular approach would be suitable to elicit practice change. Methodologically, it is not clear what the process for determining the “dialogical exchanges” was based on, particularly since there is not one reference provided regarding dialogical exchanges. It would help if the authors, even in one brief sentence, made it clear how they came up with what would be the parameters of the “dialogical exchange” or at the very least provided a reference. It would also be interesting and important to know the specific qualifications of the researchers to be providing this type of “critically reflective mentorship” (I will call it for lack of a better term) to the clinicians. The authors run the risk of coming across as somewhat paternalistic in their role as researchers to the clinicians unless they can explicate some specific set of skills that the particular researcher possesses or some specific resources that they drew on OR how they addressed this issue. I do not have access to reference #14 b/c its blinded but maybe include at least a comment that measures to cultivate trust were implemented, however should also be made explicit here.

Although the authors do address this issue in the RESULTS section under 1.2 Evaluation Question B, I think it is worthwhile adding one statement that addresses this issue in the Methods.

**Author response:** Thank you for this question about our use of the term ‘dialogical’. Yes, we have written in more detail about this elsewhere (Thille et al, 2018) including discussing Bhaktin. We have now unblinded this reference. We have added the following to the Methods to clarify this point as well as introduce some of our ‘qualifications’ for being able to provide this type of dialogue.

Page 5, lines 7-14: “We describe these exchanges as ‘dialogical’ because they differed substantively from ‘focus group’ methods; they were designed according to participatory research practices, and thus did not assume that any one perspective (clinical or research) was authoritative or complete (20). Our ability to foster dialogical exchange was enhanced by our own various disciplinary perspectives, including disability studies, bioethics, social psychology, sociology, geography, physiotherapy and medicine, which enabled dialogue without closure among the research team as well (reviewed in more detail in Thille, Gibson (20)).

**Reviewer 2 Comment 8:** The first sentence of this section states “In alignment with the critical social science underpinnings of our study, we used a realist approach to evaluate the intervention”. I think that sentence should be deleted. The philosophical underpinning of realist evaluation is scientific realism not critical realism. The authors may be referring to Bhaskar’s philosophy, but Pawson differentiated realist evaluation (and scientific realism) from Bhaskar and critical realism. The authors can refer to Pawson (2006) Evidence-based Policy: A Realist Perspective for more information, or just delete the first sentence Furthermore, ontological, and epistemological congruency with the larger study are not a prerequisite for RE.

**Author response:** Thank you, we recognise our error in the use of ‘critical realist’ in reference to RE (and thank you for the reference!). As mentioned above, our concerns were more for the theoretical alignment of the evaluation with the critical social science approach underpinning our research (not the other way around). We acknowledge the differences in theoretical underpinnings but considered there to be sufficient alignment for this purpose. In our edits to this section, we have added the following statement to clarify this:

Page 5, lines 21-23: “**To be consistent** with the critical social science underpinnings of our study, **we sought evaluation approaches that conceptually aligned with the study. After considering a number of evaluative approaches, we drew from elements of a** realist approach.”

RESULTS:
**Reviewer 2 Comment 9:** The authors did an EXCELLENT job of reporting qualitative findings - describing themes that are conceptually deep and sufficiently abstracted up from the data, then using well suited quotes judiciously to illustrate the findings. The findings are absolutely fascinating, so insightful, and so important. This section was a pleasure to read!

**Author response:** Thank you again!

**Reviewer 2 Comment 10:** I could be completely missing something BUT in the manuscript under results it states, “All clinicians at site 1 (n=21) and site 2 (n=20) agreed to participate….See Table 3 for demographics”. Then, when I go to Table 3 under Site 2 it says n=15, 75%.

**Author response:** We have double checked the reporting of our participant numbers against our demographic table (now Table 2) against these numbers. They appear to be accurate. We believe the reviewer might have been looking at the percentage of participants who identified as female (for site 2 this is indeed n=15, 75%). To avoid further confusion, we have added the total number of participants at each site at the top of the table (page 11).

**Reviewer 2 Comment 11:** Question 1.1 “For whom does the intervention work” is about outcomes. Then later in that section there is a sentence about the level of active participation was continent upon (1) clinician’s level of interest, and (2) familiarity with reflexivity. To me, based on definitions from RE, these are pre-existing characteristics of the actors or agents and should therefore be considered as part of the context required for the program to elicit outcomes.

**Author response:** Thank you. We agree, and have moved this section of text to 1.3.

**Reviewer 2 Comment 12:** A minor point but I think there is a word missing under section 3.1 (a) Changes to team process. There is a sentence that I believe should read “Extra rounds e ensure that all clients who were attended to were added…”

**Author response:** Thank you for picking up this typo. The sentence has been corrected. It now reads “Extra rounds **were added** to ensure that all clients were attended to, and there was a shift in focus from primarily discussing medical information…” (page 27, line 13).

DISCUSSION:
**Reviewer 2 Comment 13:** Lower physician engagement was referred to several times within manuscript, and the intervention seemed to potentially shift practice from a bio-medical, physician centred approach to stronger support, recognition, and utilization of the strengths of all team clinicians, primarily in addressing the human aspects of living with MD. This is really important b/c issues with physician engagement, the prioritization of physician-centred/bio-medical approaches, and the emphasis on the physician voice despite the resources of multi-disciplinary teams has been a long-standing issue in rehabilitation practice. I was very excited to read about the shift and therefore looking for the authors to comment on how the intervention can be used to address the longstanding issue and how RE contributed to understanding this important shift in a way that can be implemented by others. The authors talk about these shift in relation to their study/context, but I was looking to see how the authors thought their findings could inform advancement of knowledge regarding these persistent issues.

**Author response:** We appreciate this invitation to speak more broadly. We have added some more detail in the Discussion in response to this question about how our research might help address this persistent issue (first quote) and how it may mean that changing clinic practices are possible even if physicians are not involved (second quote).

Page 34, lines 1-13: “Our evaluation suggests that the intervention was largely successful in engaging clinicians in the human aspects of living with MD and the applications to clinical care. **This work responds to the now considerable transdisciplinary research findings, including our own, that highlight a need for a shift in focus of healthcare on biologically repairing/adapting the body to the exclusion of the human aspects of care (e.g., 34, 35-37). The interventional approach could potentially be used by other researchers, clinicians or service designers to facilitate this shift, and related persistent issues such as physician-centred approaches and underutilisation of the other resources within multidisciplinary teams. The attention that both the research methodology and evaluative approach provide to the** specifics of the context **is likely to be vital to creating a shift in such imbedded practices. This includes the** flexible, participatory design **and evaluation; diverse makeup of the investigator team (e.g.,** clinicians, **consumers** and social theorists); longitudinal timeframe; and **use of** applied critical reflexivity as both a study approach and teachable skill.

Page 35, lines 7-12: Finally, a key challenge was the engagement of physicians. We would suggest addressing the participation of any busy and/or disengaged clinicians within the project design, perhaps by using incentives, discussing it with them privately, or conducting the study contexts where physicians/clinicians already regularly attend meetings. **However, the distributed emphasis of the intervention on the human aspects of care may support shifts in clinic practices even if more biomedically-focussed clinicians are not involved.**
